# A Novel Checkpoint and RPA Inhibitory Pathway Regulated by Rif1

**DOI:** 10.1371/journal.pgen.1002417

**Published:** 2011-12-15

**Authors:** Yuan Xue, Michael D. Rushton, Laura Maringele

**Affiliations:** Institute for Ageing and Health, Newcastle University, Newcastle upon Tyne, United Kingdom; Fred Hutchinson Cancer Research Center, United States of America

## Abstract

Cells accumulate single-stranded DNA (ssDNA) when telomere capping, DNA replication, or DNA repair is impeded. This accumulation leads to cell cycle arrest through activating the DNA–damage checkpoints involved in cancer protection. Hence, ssDNA accumulation could be an anti-cancer mechanism. However, ssDNA has to accumulate above a certain threshold to activate checkpoints. What determines this checkpoint-activation threshold is an important, yet unanswered question. Here we identify Rif1 (Rap1-Interacting Factor 1) as a threshold-setter. Following telomere uncapping, we show that budding yeast Rif1 has unprecedented effects for a protein, inhibiting the recruitment of checkpoint proteins and RPA (Replication Protein A) to damaged chromosome regions, without significantly affecting the accumulation of ssDNA at those regions. Using chromatin immuno-precipitation, we provide evidence that Rif1 acts as a molecular “band-aid” for ssDNA lesions, associating with DNA damage independently of Rap1. In consequence, small or incipient lesions are protected from RPA and checkpoint proteins. When longer stretches of ssDNA are generated, they extend beyond the junction-proximal Rif1-protected regions. In consequence, the damage is detected and checkpoint signals are fired, resulting in cell cycle arrest. However, increased Rif1 expression raises the checkpoint-activation threshold to the point it simulates a checkpoint knockout and can also terminate a checkpoint arrest, despite persistent telomere deficiency. Our work has important implications for understanding the checkpoint and RPA–dependent DNA–damage responses in eukaryotic cells.

## Author Summary

Here we identified arguably the first anti-checkpoint protein in Rif1. The term anti-checkpoint was proposed by Ted Weinert, one of the parents of the checkpoint concept, to describe a factor that stops checkpoint proteins from responding to DNA damage by other means than repair, reduced amounts of ssDNA, or adaptation [1]. No such factor has been clearly identified; potential candidates (for example, shelterin or CST complexes at chromosome ends) may reduce the amount of damage, therefore exerting an indirect “anti-checkpoint” function. Interestingly, mammalian Rif1 was once thought to be a checkpoint protein [2]. Our study indicates that Rif1 out-competes checkpoint proteins for their substrate and sets a threshold for checkpoint activation in budding yeast. Rif1 can tune down the checkpoint responses, thus permitting cells to proliferate with DNA damage, a pre-requisite for chromosomal instability, the hallmark of cancer cells. Rif1 is an important link in understanding how eukaryotic cells balance the need to proliferate with the need to preserve their genetic heritage. Finding an anti-checkpoint is not of pure theoretical interest. In the future, Rif1 inhibitors could limit proliferation of chromosomally unstable cells. Conversely, Rif1 enhancers could tune down the over-blown checkpoint responses that lead to massive cell death following different insults.

## Introduction

Telomeres protect chromosome ends from activating DNA damage responses that result in cell cycle arrest or inadvertent “repair.” Evidence that telomere dysfunction could be involved in carcinogenesis [3]–[5] suggests that some telomere-defective cells are able to avoid/escape arrest and generate genetically modified progenies. Checkpoint inactivation and checkpoint adaptation are potential routes to escape from arrest. Checkpoint adaptation is an intriguing process, in which checkpoint responses are terminated, despite persistent DNA damage and intact checkpoint pathways (reviewed by [6]). Several proteins involved in checkpoint adaptation have been identified. However, many if not all (Ku, Mre11, Rad50, Tid1, Srs2, Sae2, Cdc5^Polo^) also participate in processing the DNA damage [7]–[12]. Therefore, these proteins permit escape from arrest most likely indirectly, by affecting the substrate required for checkpoint activation.

Exciting discoveries in model organisms as diverse as *Schizosaccharomyces pombe* and *Drosophila melanogaster* suggest that eukaryotic cells are quite resourceful in their ways to prevent chromosome ends from being detected as DNA damage. For example, dysfunctional *S. pombe* telomeres do not recruit the checkpoint protein Crb2^53BP1^, most likely because they lack a particular checkpoint substrate [13]. Drosophila uses transposons to protect and maintain chromosome ends, whereas *S. pombe* can use ribosomal DNA for the same purpose, but only when telomerase is inactivated [14]. In contrast, *Saccharomyces cerevisiae* can proliferate without transposons, telomeric or ribosomal DNA at chromosome ends when telomere maintenance pathways, e.g. telomerase and telomere recombination, are inactivated [15]. Such budding yeast strains, called PAL survivors, have no particular DNA sequences at chromosome ends, which shorten progressively, without triggering a cell cycle arrest. [15]. The existence of the PAL survivors suggests that eukaryotic cells can also prevent checkpoint responses to chromosome ends in a sequence-independent manner, perhaps with help from “anti-checkpoint” factors.

In this study, we identified arguably the first “anti-checkpoint” protein in Rif1 and demonstrate that checkpoint responses to damaged chromosome ends can be inhibited without significant modification of a major checkpoint substrate, the single stranded DNA. We propose that Rif1 has important physiological roles in preventing a cell cycle arrest to incipient or small single stranded DNA lesions occurring on chromosomes, particularly on chromosome ends. However, high levels of Rif1 may contribute to genomic instability by facilitating cell proliferation with even more DNA damage.

## Results

### Rif1 associates with DNA damage differently from Rap1

Telomeres successfully avoid stimulating the DNA damage checkpoint pathways, despite their resemblance to broken chromosome ends. Therefore, we hypothesized that proteins able to inhibit checkpoint sensors are among the telomere-associated proteins. To unmask potential checkpoint inhibitors, we tested how Rap1, Rif1 and other telomere-associated proteins respond to DNA damage. Rap1 is a major component of the telomeric chromatin [16], whereas Rif1 is a Rap1-interacting factor [17]. To induce DNA damage, we used the well-studied model system *cdc13-1*. Budding yeast *cdc13-1* cells have a temperature-sensitive mutation in the telomere capping protein Cdc13^Pot1^. At restrictive temperatures, telomeres become uncapped and vulnerable to DNA processing factors. Hence, Sgs1 and other helicases unwind telomeres [18], whereas Exo1 and other nucleases resect the 5′-ended DNA strand. Together, they generate single stranded DNA (ssDNA), a potent checkpoint activator.

**Figure 1:**
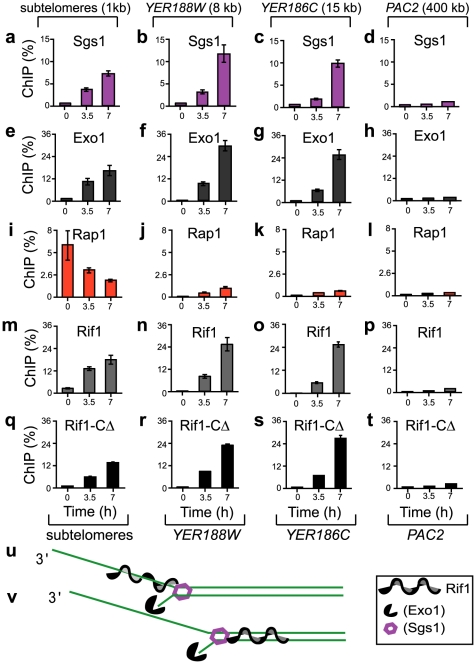
The association of Sgs1, Exo1, Rap1, Rif1, and Rif1-C*Δ* with different chromosome regions during telomere dysfunction. All strains were *cdc13-1*, grown overnight at 21°C, followed by 7 hours at 36°C to induce telomere uncapping. “ChIP (%)” was calculated for each sample (time-point) as the difference between the immuno-precipitated DNA and the background, divided by input DNA, and multiplied by 100. Error bars represent the standard deviation (SD) between three measurements. (a–d) The recruitment of Sgs1 to chromatin at the following distance from chromosome ends: (a) 1 kb (in Y′ sub-telomeric repeats) (b) 8 kb (in *YER188W*), (c) 15 kb (in *YER186C*) and (d) 400 kb (in *PAC2*). *YER188W, YER186C* and *PAC2* are single gene loci on chromosome 5. Analyzed chromosome regions are indicated at the top of the figure. (e–h) As in a–d, except that Exo1 was analyzed; (i–l) Rap1 was analyzed; (m–p) Rif1 was analyzed; (q–t) Rif1-C*Δ* (1-1350 amino acids) was analyzed. Analyzed proteins are indicated above each graph. Exo1, Rif1 and Rif1-C*Δ* are Myc-tagged. Sgs1 and Rap1 are not tagged. (u–v) Preliminary models of interaction between Rif1 and resection forks: (u) Rif1 associates with ssDNA accumulating behind resection forks; (v) DNA unwinding pushes Rif1 ahead of the resection fork.

To determine whether the association of Rif1 and Rap1 with chromosomes was affected by the recruitment (and subsequently by the activities) of Sgs1 and Exo1, we induced telomere uncapping by shifting *cdc13-1* cells from permissive (21°C) to restrictive (36°C) temperature. The dynamics of Sgs1 recruitment to uncapped chromosome ends is not known. Using chromatin immuno-precipitation, we found that Sgs1 did not significantly associate with sub-telomeres at 21°C (time 0 in Figure 1a). At the restrictive temperature 36°C however, Sgs1 progressively accumulated at 1, 8 and 15 kb from chromosome ends, associating with (sub)telomeres and single gene loci, whereas it did not associate with the centromere-proximal *PAC2* locus (Figure 1a–1d). Exo1 accumulated at the same regions and with similar dynamics to Sgs1 (Figure 1e–1h), suggesting that: 1) DNA unwinding is closely followed by an Exo1-dependent resection and 2) with time, increasingly more sub-telomeres and single gene loci are processed by Exo1/Sgs1.

Under these conditions, we also determined the dynamics of Rap1 and Rif1 onto DNA. We found that both Rap1 and Rif1 associated with (sub)telomeres at 21°C (time 0, Figure 1i, 1m), consistent with other studies [19], [20]. At the restrictive temperature 36°C however, Rap1 and Rif1 behaved differently from each other. Whereas Rap1 progressively dissociated from (sub)telomeres, Rif1 accumulated in (sub)telomeres (Figure 1i, 1m). Whereas only small levels of Rap1 (below 1%) were detected at single gene loci, 8 and 15 kb from the right end of chromosome 5 (Figure 1j–1k), high levels of Rif1 (up to 26%) were detected at these loci (Figure 1n–1o). In conclusion, Rif1 associated similarly to Sgs1 and Exo1; two different models of Rif1 association with DNA damage are presented in Figure 1u–1v. Moreover, Rif1 associated differently from Rap1. This is surprising, since it was thought that Rif1 associates with DNA through Rap1 [17].

To confirm that Rif1 does not require Rap1 to associate with DNA damage, we generated *cdc13-1* strains lacking the C-terminus of Rif1 (Rif1-C*Δ*), required for association with Rap1 [17]. We found that Rif1-C*Δ* did not significantly associate with sub-telomeres at 21°C (Time 0 in Figure 1q), suggesting that Rif1 requires Rap1 to be recruited to normal (sub)telomeres, consistent with other studies [17]. Following telomere uncapping however, Rif1-C*Δ* progressively associated with sub-telomeres and single gene loci (Figure 1r–1t), similarly to Rif1 (and different from Rap1). These data indicate that Rif1 is recruited to DNA damage independently of Rap1. Interestingly, the behaviour of Rif1-C*Δ* bears a striking resemblance to that of mammalian Rif1 [2], [21], suggesting conserved functions. Similarly to Rif1-C*Δ*, mammalian Rif1 does not co-localize with Rap1 at normal telomeres, however it associates with dysfunctional telomeres and other damaged regions [2], [21].

### Rif1 inhibits the association of Rad9^53BP1^, Ddc1^Rad9^, Ddc2^ATRIP^, and RPA with DNA damage (DNA junctions)

**Figure 2:**
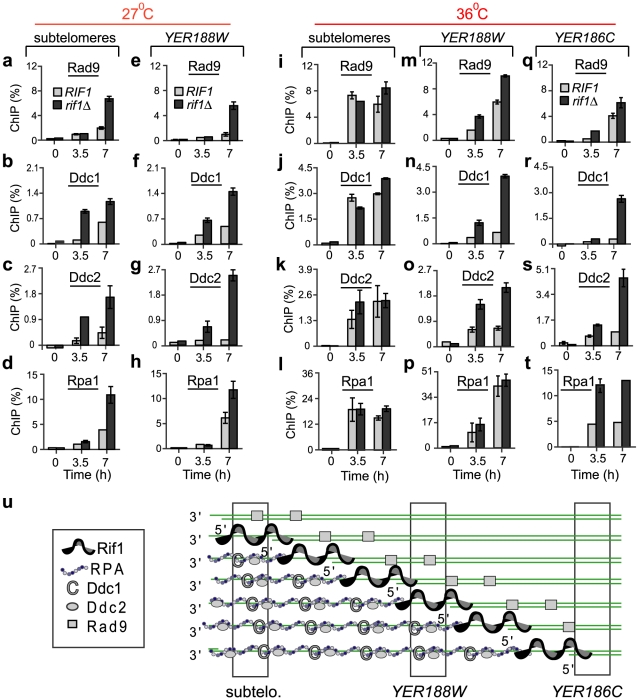
Rif1 inhibits the recruitment of checkpoint proteins and Rpa1 to sub-telomeres and single gene loci. *Cdc13-1* cells with a *rif1Δ* mutation (dark columns) or with wild-type *RIF1* (light columns) were grown overnight at 21°C, followed by for 7 hours at 27°C or 36°C to induce telomere uncapping. ChIP (%) was calculated as in Figure 1. Analyzed chromosome regions are indicated at the top of the figure. (a–h) The dynamics of Rad9, Ddc1, Ddc2 and Rpa1 association with chromosome ends at 27°C. (i–t) The dynamics of Rad9, Ddc1, Ddc2 and Rpa1 association with chromosome ends at 36°C. Each analyzed protein is indicated above the respective graph. Ddc1 is HA-tagged; Rpa1 and Ddc2 are YFP-tagged; Rad9 is not tagged. Time 0 temperature and error bars are as in Figure 1. (u) A model of Rif1-association with excised chromosome ends in a population of cells. Vertical bars indicate regions analyzed in a–t.

At DNA damage, Rif1 may occupy the substrate for important DNA damage response (DDR) proteins. Ddc1^Rad9^ (part of the 9-1-1 checkpoint complex) has high affinity for 5′-junctions between single and double stranded DNA [22]. The RPA complex has high affinity for ssDNA, facilitating the recruitment of the Ddc2^ATRIP^ checkpoint mediator [23], [24]. Rad9^53BP1^ checkpoint protein associates with chromatin adjacent to DNA damage. Therefore, we determined whether Rif1 interfered with the association of any of these proteins with sub-telomeres and single gene loci. We used the same experimental design as in Figure 1, except that we incubated *cdc13-1* cells at their mildest restrictive temperature (27°C). Interestingly, we found that several fold more checkpoint protein (Rad9^53BP1^, Ddc1^Rad9^ and Ddc2^ATRIP^) and RPA associated with (sub)telomeres and *YER188W* in *rif1D* than in *RIF1+* cells, during 7 h at 27°C (Figure 2a–2h), indicating that Rif1 strongly inhibits their recruitment to DNA damage.

**Figure 3:**
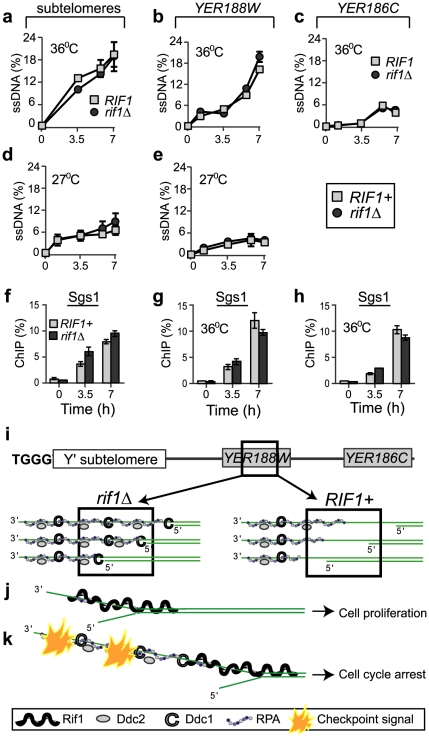
Rif1 does not inhibit resection of sub-telomeres and single gene loci. (a–c) Quantification of ssDNA generated in *cdc13-1* cells with a *rif1Δ* mutation (dark circles) or with wild-type *RIF1* (light squares) at 36°C at: (a) 1 kb, in sub-telomeres, (b) 8 kb, in *YER188W* and (c) 15 kb, in *YER186C*. (d–e) As in a–b, except that the temperature was 27°C. We used QAOS (the Quantitative Amplification Of ssDNA) to detect ssDNA as in [29], [51]. (f–h) The dynamics of Sgs1 association with chromosome ends following telomere uncapping at 36°C, in *cdc13-1* cells with a *rif1Δ* mutation (dark columns) or with wild-type *RIF1* (light columns) at: (f) 1 kb, in sub-telomeres, (g) 8 kb and (h) 15 kb. Time 0 temperature and error bars are as in Figure 1. (i) A model of RPA and checkpoint proteins association with *YER188W* in *rif1Δ* (left) versus *RIF1+* cells (right). Squares indicate chromosomal regions detected by ChIP and QAOS. (j–k) The ssDNA tolerance threshold model: (j) When short ssDNA lesions are generated, Rif1 acts as a “band-aid” for these lesions, associating with DNA-junctions and the adjacent ssDNA and blocking access for RPA and checkpoint proteins. In consequence, cells avoid spending energy to arrest and to re-start the cell cycle. (k) When longer ssDNA lesions are generated, they extend beyond Rif1-protected regions. In consequence, the damage is detected, a checkpoint signal is fired and cells arrest the cell cycle.

To inhibit checkpoint proteins and RPA, Rif1 may associate with ssDNA at random, similarly to RPA, or mainly with DNA-junctions and the adjacent (ss)DNA. To distinguish between these possibilities, we analyzed the effect of Rif1 at 36°C. At this temperature, resection is faster and affects many more chromosomes than at 27°C (see Figure 3). Consequently, DNA-junctions move faster from (sub)telomeres towards internal regions like *YER188W* and *YER186C*, leaving behind long ssDNA overhangs, bound by RPA and checkpoint proteins (Figure 2u). Therefore, we determined the location of Rif1 as follows: 1) If Rif1 associates with ssDNA at random, the Rif1-effect will be region-independent; 2) If Rif1 associates with DNA-junctions and the adjacent DNA, the Rif1-effect will appear stronger towards internal regions and weaker or absent towards (sub)telomeres. Importantly, we detected a strong Rif1-inhibitory effect on checkpoint/RPA proteins at *YER188W* and/or *YER186C* loci (Figure 2m–2t). Conversely, no such Rif1-effect was detected in sub-telomeres (Figure 2i–2l). These data strongly suggests that Rif1 associates with DNA damage at or around DNA-junctions (Figure 2u).

### Rif1 does not affect resection of sub-telomeres or single gene loci

The ability of proteins like Cdc13 and KU to inhibit the recruitment of checkpoint proteins and/or RPA to DNA is indirect, by inhibiting DNA resection [25]. One possibility is that the Rif1-effect is also indirect. A recent study used in-gel hybridization assays to claim there is more ssDNA in *cdc13-1 rif1Δ* versus *cdc13-1* cells at the very end of the chromosomes, the TG-telomeric sequences [26]. However, no other chromosomal regions were investigated. Because the in-gel hybridization assay is not sensitive enough for sub-telomeres or single gene loci, we used the ultra-sensitive qPCR-based method QAOS [27]–[29] to test whether a difference in ssDNA was responsible for the different accumulation of RPA and checkpoint proteins in *RIF1+* versus *rif1Δ* cells. We found that this was clearly not the case. Unlike its strong inhibitory effect on RPA and checkpoint proteins, Rif1 did not significantly inhibit the accumulation of ssDNA in sub-telomeres or single gene loci, because similar amounts were detected in either *rif1Δ* or *RIF1* cells at different time points during 7 h at 36°C or 27°C (Figure 3a–3e). Moreover, Rif1 did not affect the dynamics of the Sgs1 helicase on chromosome ends (Figure 3f–3h). Therefore, the inhibitory Rif1-effect on checkpoint proteins and RPA was not through inhibiting ssDNA formation.

In summary, several fold more RPA/checkpoint protein associated with sub-telomeres and single gene regions in *rif1Δ* versus *RIF1* cells, despite the fact that very similar amounts of ssDNA accumulated at those regions in *rif1Δ* and *RIF1* cells (Figure 3i). To our knowledge, this is the first report of a protein inhibiting the recruitment of RPA and checkpoint proteins to a chromosome region, without inhibiting resection or unwinding of that region. Rif1 most likely occupies the DNA junction and the adjacent DNA, thus blocking access for other proteins (e.g. checkpoint and RPA) to these important DDR structures. Through this effect, we propose that Rif1 sets the ssDNA tolerance threshold, facilitating cell proliferation with lower amounts of ssDNA (limited to regions protected by Rif1) or permitting arrest, if ssDNA is more extensive (Figure 3j, 3k). We tested this hypothesis by investigating the effect of Rif1 on cell proliferation and checkpoint responses, discussed next.

### Rif1 inhibits the checkpoint responses to “below-threshold” ssDNA

**Figure 4:**
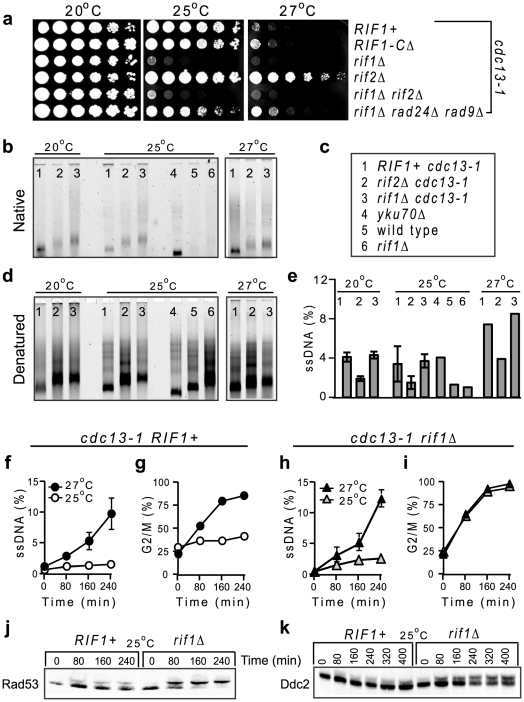
Rif1 inhibits the checkpoint responses to telomere uncapping in *cdc13-1* cells. (a) Growth of serial dilution of *cdc13-1* cells with or without additional mutations (indicated at the right of each row) at different temperatures, indicated above each plate. (b–e) Over-night cultures at 20°C were incubated with nocodazole for 2 h and then shifted to 25°C or to 27°C for 3 h. DNA was digested and hybridized with a CA-rich probe. (b) TG signals at native (non-denatured) telomeres. Numbers at the top of each lane correspond to different mutants, revealed in c. (c) Legend for b, d, and e indicating relevant mutations. (d) TG signals at denatured telomeres. (e) The percentage of ssDNA represents the fraction of native TG-ssDNA normalized to the total of TG sequences in each denatured sample. Error bars represent the standard deviation between measurements from two independent experiments. (f–g) The ssDNA was quantified by QAOS and the G2/M fraction counted by microscopy in *cdc13-1 RIF1+* cells incubated for 240 min at 25°C (white circles) or 27°C (black circles). (h–i) As in f–g, except that cells were *cdc13-1 rif1Δ* at 25°C (white triangles) or 27°C (black triangles). (j) Rad53 activation detected by western blotting in *cdc13-1* cells with wild type *RIF1* (left) or with a *rif1Δ* mutation (right), incubated at 25°C, (except for the time 0 when the temperature was 21°C). (k) As in (j), except that the Ddc2 activation was detected.

To test whether Rif1 affects the ssDNA tolerance threshold, we investigated how much ssDNA could *rif1Δ* cells tolerate without arresting in G2/M, compared to *RIF1+* cells. To generate low amounts of ssDNA, we incubated *cdc13-1* cells (+/- other relevant mutations) at the permissive temperature of 25°C. To generate more ssDNA, we incubated *cdc13-1* cells at 27°C. Consistent with the idea that Rif1 facilitates cell proliferation with low amounts of ssDNA and also with some recent observations [26], [30], a *rif1Δ* mutation was incompatible with proliferation of *cdc13-1* cells at 25°C (Figure 4a). In contrast, *cdc13-1* cells with a C-terminal truncated version of Rif1 (Rif1-C*Δ*) proliferated as well as those with the wild type Rif1 (Figure 4a). This indicates that the interaction between Rif1 and Rap1 (disrupted in the truncated version) is not relevant for facilitating proliferation of telomere-uncapped cells, consistent with our observation that Rif1-C*Δ* continues to associate with DNA damage (Figure 1q–1t).

Since *rif1Δ cdc13-1* cells had longer telomeres than *cdc13-1* cells (about 0.6 kb versus 0.3 kb), we asked whether their enhanced temperature-sensitivity was somehow related to telomere length. Therefore, we tested a *rif2Δ* mutant, which has also longer telomeres [31]. In contrast to a *rif1Δ* mutation, a *rif2Δ* mutation permitted *cdc13-1* cells to proliferate at 25°C and even at 27°C. Strikingly, deletion of *RIF1* in *cdc13-1 rif2Δ* cells rendered them unable to proliferate at 25°C (Figure 4a), indicating a dominant effect for *rif1Δ*. Deletion of *RAD24* and *RAD9* checkpoint genes partially rescued the proliferation of *cdc13-1 rif1Δ* cells (Figure 4a). In conclusion, Rif1 is essential for proliferation of *cdc13-1* and *cdc13-1 rif2Δ* cells at 25°C and 27°C respectively, through a mechanism independent of telomere length.

A recent report by Anbalagan *et al* attributed the increased temperature-sensitivity of *cdc13-1 rif1Δ* cells to increased ssDNA in the TG-rich telomeric repeats [26]. This interpretation is slightly at odds with our findings that Rif1 did not affect ssDNA accumulation at 0.6 kb distance from telomeres (in sub-telomeres) or at more internal regions, at 27°C and 36°C (Figure 3). It implies that Rif1 inhibits a very unusual resection activity that is restricted to TG-telomeres or to temperatures around 25°C. However, Anbalagan *et al* have not quantified and normalized the TG-signals to the total amount of TG sequences. It is known that *rif1Δ* cells have up to four times more TG sequences (longer telomeres) than *RIF1+* cells. Therefore, normalizing the TG-signals is essential for determining whether stronger signals are caused by more ssDNA, or merely by more TG-sequences *per* ssDNA kilobase. To find out which one is true, we used similar techniques and experimental conditions as in Anbalagan *et al*., except that we quantified and normalized the original TG-ssDNA signals to the total amount of TG repeats, used a previously described AC-rich fluorescent probe [32], analyzed a *cdc13-1 rif2Δ* strain as control for signals generated by longer TG-telomeres and tested an additional temperature (27°C).

To determine whether Rif1 affects telomere resection at 25°C, we needed to compare ssDNA accumulation in cells expected to arrest proliferation in G2/M (*cdc13-1 rif1Δ*) versus cells expected to divide (*cdc13-1* +/-*rif2Δ*). However, ssDNA lesions at *cdc13-1* uncapped telomeres are generated almost exclusively in the G2/M phase [33]. Moreover, DNA replication factors would most likely repair (re-synthesize) some of the lesions during S-phase, if cells could proliferate. Therefore, nocodazole was used to arrest all strains in G2/M, thus permitting comparison between their resection rates based upon genetic differences, rather than cell cycle differences. Over-night cultures at 20°C were incubated with nocodazole for 2 h and then shifted to 25°C or to 27°C for 3 h. The signals given by TG-ssDNA (e.g. ssDNA hybridized with an AC-rich probe) at native and denatured chromosome ends are shown in Figure 4b and 4d, respectively. The TG-ssDNA signals normalized to the total amount of TG repeats are presented in Figure 4e.

In these experiments, the wild type and *rif1Δ* controls accumulated only about 1% TG-ssDNA, whereas strains with mutations in the telomere-capping proteins Cdc13 or Yku70 accumulated more ssDNA. However, we found similar fractions of ssDNA at telomeres of *cdc13-1* and *cdc13-1 rif1Δ* cells: about 4% at 20–25°C and 7–8% at 27°C (Figure 4e). The error bars (the standard deviation between measurements from two different experiments) are also overlapping. This shows that Rif1 does not significantly affect resection of telomeres, consistent with similar results we obtained at other chromosome regions and temperatures using QAOS (Figure 3 and Figure 4). In contrast, ssDNA was two-fold lower in *cdc13-1 rif2Δ* versus *cdc13-1* cells at any tested temperature (Figure 4e). Together, these data suggest that Rif1 and Rif2 have different effects: Rif1 does not affect resection, whereas Rif2 somehow facilitates resection at telomeres.

Interestingly, strains able (*cdc13-1* and *yku70Δ*) or unable (*cdc13-1 rif1Δ*) to proliferate at 25°C had similar fractions of telomeric TG-ssDNA (about 4%) at this temperature (Figure 4e). Moreover, *cdc13-1 rif1Δ* cells had similar fractions of telomeric ssDNA at permissive (20°C) and restrictive (25°C) temperatures (Figure 4e). These data indicate that resection of TG-telomeres does not correlate with cell cycle arrest. Therefore, we tested whether progression of ssDNA to sub-telomeres correlated with G2/M arrest. We incubated *cdc13-1* strains (+/- other relevant mutations) at 25°C and 27°C, without nocodazole. We found that about 2% ssDNA accumulated in *RIF1+* cells at 25°C (Figure 4f), however most cells continued to cycle (Figure 4g) and did not activate the Rad53^Chk2^ checkpoint kinase (Figure 4j) or the Ddc2^ATRIP^ checkpoint protein (Figure 4k). At 27°C, about 6–10% ssDNA was detected in sub-telomeres when over 75% *RIF1+* cells accumulated in G2/M (Figure 4f–4g). These data suggest that the majority of *RIF1+* cells arrest proliferation in response to about 6–10% sub-telomeric ssDNA, whereas they tolerate 2% sub-telomeric ssDNA without activating a checkpoint response.

In contrast, *rif1Δ* cells responded to about 2% sub-telomeric ssDNA at 25°C by accumulating in G2/M as fast as they did in response to higher ssDNA levels at 27°C (Figure 4h–4i). A 2% sub-telomeric ssDNA corresponds roughly to one chromosome end *per* cell being resected as far as 1 kb. Another 1–2 telomeres are also likely to be single-stranded (since 4% ssDNA was detected in telomeres, Figure 4e), bringing the estimated total ssDNA to about 2 kb *per* cell. This is below the 10 kb ssDNA threshold predicted to activate checkpoints [10], [34]. These data indicate that Rif1 regulates the threshold for checkpoint activation in response to ssDNA, thus permitting proliferation with DNA damage.

### High level of Rif1 expression mimics a checkpoint knockout

**Figure 5:**
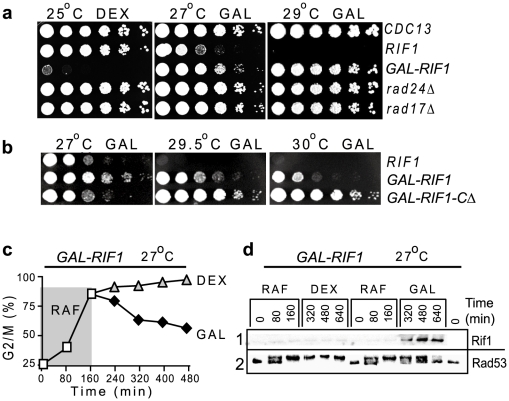
Rif1 over-expression simulates a checkpoint knockout; Rif1-induction terminates a checkpoint response. (a) Growth of serial dilution of wild type (first row, marked as *CDC13*) and *cdc13-1* cells with *RIF1* under its own promoter (second row) or under the *GAL1* promoter (third row) or with additional mutations (indicated on the right of each row). (b) Growth of serial dilutions of *cdc13-1* cells with *RIF1* under its own promoter (first row); with *RIF1* under the *GAL1* promoter (second row); with the truncated *RIF1-CΔ* version under the *GAL1* promoter. Temperatures and sugars in the medium are indicated above each plate: DEX stands for dextrose, GAL for galactose. (c) A culture of *cdc13-1 GAL1-GFP-RIF1* cells was grown on raffinose (RAF) for 160 min at 27°C, then divided in two sub-cultures and either dextrose or galactose added; white squares represent the percentage of G2/M cells on RAF; triangles on DEX; black rombi on GAL. (d) Western blotting with anti-GFP to detect expression of GFP-Rif1 (lane 1) or with anti-Rad53 (lane 2) in samples collected during the experiment described in c.

If Rif1 is a major ssDNA threshold-setter, then this threshold may increase with increased Rif1 expression. To test this hypothesis, we over-expressed *RIF1* from the *GAL1* promoter, induced by galactose. We found that Rif1 over-expression permitted *cdc13-1 GAL*-*RIF1* cells to proliferate at 27°C and even at 29°C, similarly to the effect of a *rad24Δ* or a *rad17Δ* mutation (Figure 5a). Rad24^Rad17^ and Rad17^Rad9^ are checkpoint proteins, essential to arrest the cell cycle of telomere-damaged *cdc13-1* cells [25]. Thus, Rif1 over-expression has the same effect as a checkpoint knockout, eliminating cell cycle arrest.

Interestingly, the over-expression of the C-terminal truncated variant *RIF1-CΔ* had additional effects on proliferation of *cdc13-1* cells, increasing their temperature-resistance even further, to 30°C (Figure 5b). However, *cdc13-1 GAL-RIF1-CΔ* proliferated slower than *cdc13-1 GAL-RIF1* on galactose (compare their growth at 27°C, Figure 5b). One hypothesis explaining these apparently paradoxical effects could be that an excess of *RIF1-CΔ* slows down DNA replication, which in turn allows more time for DNA replication factors to repair (re-synthesize) some of the ssDNA lesions at uncapped telomeres. Therefore, cells divide slower, but tolerate DNA damage better. Although we have not investigated the effects of over-expressing *RIF1-CΔ* any further, such mutants may be useful to determine whether Rif1 plays a role in other cellular processes like DNA replication.

### Induction of Rif1 can terminate a checkpoint response

Another plausible consequence of the anti-checkpoint effect could be that Rif1 is able to terminate a checkpoint response when induced in cells that have already accumulated DNA damage. To test this hypothesis, we grew *cdc13-1 GAL-RIF1* cells on raffinose at 27°C, to induce DNA damage and cell cycle arrest (Figure 5c). Then, we added either dextrose or galactose to the cultures. We found that with time, increasingly large fractions of *GAL-RIF1* cells escaped from arrest in galactose, but not in dextrose (Figure 5c). Since galactose stimulates expression of Rif1, whereas dextrose inhibits it, this indicates that Rif1 over-expression abolishes a cell cycle arrest. Escape from arrest was confirmed by a progressive reduction in the active form of Rad53^Chk2^ (Figure 5d). In conclusion, (over-expressed) Rif1 was able to terminate a checkpoint-dependent arrest caused by a telomere uncapping.

The fact that over-expressed Rif1 inhibited the recruitment and activation of checkpoint proteins to the point it emulated a checkpoint knockout is consistent with the model presented in Figure 3j–3k, in which Rif1 out-competes the checkpoint proteins for their substrate. The fact that several hours after its induction, Rif1 was able to terminate a checkpoint arrest in a large fraction of cells, suggests that Rif1 took advantage of the turnover of RPA/checkpoint proteins on DNA damage to occupy their substrate of detection. Additionally, we suggest that Rif1 could be recruiting phosphatases to the DNA damage, to de-activate checkpoint proteins. Consistent with this hypothesis, Rif1 interacts physically with four different phosphatases: Glc7, Cdc14, Ptp1 and Psr2 [35]. Future studies will determine whether phosphatases are required for the Rif1-effect. In conclusion, Rif1 has an anti-checkpoint, anti-RPA effect at natural, but dysfunctional chromosome ends. This raises the question of whether Rif1 has similar effects at the ends of a double strand break (DSB).

### Rif1 does not associate with an HO-DSB

**Figure 6:**
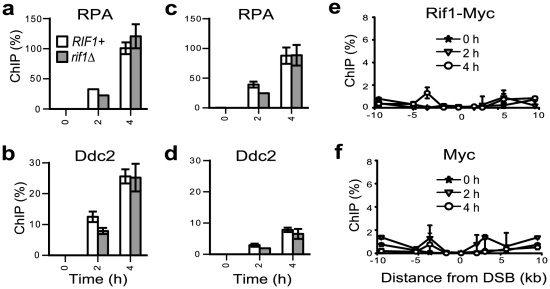
Rif1 does not associate with an HO-induced DSB. Strains were grown overnight on raffinose; galactose was added to the medium at time 0 and samples collected every 2 hours. Galactose induces GAL-HO-nuclease to cut an unique DSB at the MATa locus [7]. Although a DSB is usually repaired by recombination, recombination was prevented in JKM139-derived strains by deletion of the donor locus [7]. (a–b) Chromatin immuno-precipitation experiments indicating the association of RPA and Ddc2 with chromatin at 0.2 kb right from the DSB, in *RIF1+* (white columns) and *rif1Δ* (grey columns) JKM139-derived strains. (c–d) As in a–b, except that the association of RPA and Ddc2 with chromatin at 2 kb right from the DSB was analyzed. e) Chromatin immuno-precipitation with Myc antibody in the Rif1-Myc JKM139-derived strain. Legend indicates the time in galactose. The numbers on the X-axis indicate the location of the DSB (marked as 0) and the distance from the DSB (in kilobases). (f) As in (e), except that immuno-precipitation with Myc antibody was performed in the untagged JKM139 strain.

A DSB poses an immediate threat to the viability of cells; in response to a DSB, cells arrest proliferation within one single cell cycle [36], [37]. Therefore, it is perhaps unlikely that checkpoint inhibitors would be acting at a DSB. To test this hypothesis, we induced the HO nuclease to cut a DSB (at a locus where it could not be repaired by homologous recombination) as previously described [7] and compared the recruitment of RPA and Ddc2^ATRIP^ in *RIF1+* versus *rif1Δ* strains. We found that Rif1 did not significantly affect the association of RPA or Ddc2^ATRIP^ with chromosomal DNA at 0.2–2 kb distance from the DSB (Figure 6a–6d). We investigated the reasons behind this lack of effect and found that Rif1 was not significantly recruited to a DSB (Figure 6e–6f). This result reinforces the idea that Rif1 has to associate with DNA damage, in order to inhibit RPA/checkpoint proteins.

## Discussion

Our study indicates that Rif1 has a novel anti-RPA, anti-checkpoint effect. This effect manifested through less RPA and checkpoint protein recruited in *RIF1+* versus *rif1Δ* cells to DNA damage (ssDNA) caused by telomere uncapping. Normal expression of Rif1 prevented a checkpoint-dependent cell cycle arrest in response to relatively low levels of ssDNA (estimated at 2–10 kb ssDNA *per* cell), whereas Rif1 over-expression prevented/abrogated a cell cycle arrest in cells with higher levels of ssDNA, similarly to the effect of a checkpoint knockout. Therefore, we proposed that Rif1 has a physiological role in regulating the checkpoint activation threshold in response to ssDNA; our model is presented in Figure 3i–3k.

We also investigated the mechanism(s) by which Rif1 inhibits checkpoint proteins/RPA and found that: 1) Rif1 did not significantly affect the amount of ssDNA at any chromosomal region (Figure 3, Figure 4); 2) Rif1 did not affect the amount of checkpoint protein in cells (Figure S1); 3) Rif1 inhibited the recruitment of checkpoint proteins/RPA when Rif1 associated with their DNA substrate (Figure 1, Figure 2, Figure 3). Conversely, Rif1 had no effect on checkpoint proteins/RPA, when Rif1 did not associate with their DNA substrate (Figure 6). These data strongly suggest that Rif1 inhibits the recruitment of checkpoint proteins/RPA to DNA damage through a competitive inhibition mechanism. Clues about the conditions required for Rif1 to out-compete RPA and checkpoint protein could be found in the different behavior of Rif1 at chromosome ends versus internal double strand breaks, discussed next.

Several hypotheses could explain the absence of Rif1 from an internal DSB: 1) Putative recruiters of Rif1 are missing from a DSB; 2) Rif1 is out-competed by other proteins. 3) Rif1 associates only with DNA damage initiated from telomeres. Although we have not pursued these hypotheses, our favourite is the second one. This is because a recent study showed that the majority of Rif1 is bound to the nuclear membrane [38], the place where telomeres are usually anchored. Therefore, we propose that Rif1 can efficiently out-compete other proteins for damages occurring near regions with abundant Rif1 (e.g. close to the nuclear periphery and/or to telomeric or sub-telomeric sequences). Conversely, Rif1 is usually out-competed at DNA damage occurring further from Rif1 anchor sites. Protein(s) preventing Rif1 from binding an internal DSB may also prevent checkpoint proteins from detecting lower amounts of ssDNA (caused by incipient resection/unwinding of the DSB), thus setting a Rif1-independent checkpoint-activation threshold. These do exclude the possibility that Rif1 may associate with internal DSBs under certain conditions, which remain to be investigated.

The association of Rif1 with chromatin within 15 kb from chromosome ends was demonstrated by chromatin immuno-precipitation in experiments presented in Figure 1. Interestingly, we found that Rif1 associated with DNA damage differently from Rap1 and that Rif1 also associated in the absence of its C-terminal Rap1-binding domain (Figure 1). If Rap1 was not required, perhaps other proteins facilitated the recruitment of Rif1 to DNA damage. Similarly to Rif1, a putative recruiter should associate with DNA-junctions and/or resection forks. Among candidates with these characteristics are the Rad24^Rad17^ checkpoint clamp loader and the Sgs1 helicase. Rad24^Rad17^ recruits Ddc1^Rad9^ (a protein investigated in Figure 2) as part of the 9-1-1 complex. If Rif1 has high affinity for Rad24^Rad17^, it will competitively inhibit Ddc1^Rad9^/9-1-1, thus providing an alternative explanation for some of the anti-checkpoint Rif1-effects. However, we found no evidence that Rad24^Rad17^ or Sgs1 are recruiting Rif1 (Figure S1, discussed in Text S1). The fact that Rif1 had a much stronger effect when over-expressed (without the need to over-express a potential recruiter) may suggest that Rif1 does not require a recruiter to DNA damage.

Previous studies suggested that Rif1 and Rif2 inhibit telomerase [31], [39], [40], an enzyme that requires a short ssDNA overhang and the activities of MRX and Tel1 to load onto DNA [41], [42]. Since Rif2 was found to inhibit an MRX-dependent DNA resection and the association of Tel1 with DNA damage [43], [44], this might explain how Rif2 inhibits telomerase. However, it cannot explain why Rif1 is a stronger telomerase-inhibitor than Rif2 (*rif1Δ* have longer telomeres than *rif2Δ* cells), since Rif1 has a much weaker effect on MRX/Tel1 compared to Rif2 [43], [44]. Our study shows that Rif1 associates with ssDNA overhangs at uncapped telomeres and protects them from RPA and checkpoint proteins. Similarly, Rif1 may associate with ssDNA overhangs generated during S-phase at normal telomeres and hide them from telomerase.

Although a molecular function for Rif1 is yet to be established in higher organisms, our study suggests conserved functions from yeast to man. We found that yeast Rif1 facilitates proliferation of cells with dysfunctional telomeres; other studies showed that mammalian Rif1 may facilitate the proliferation/viability of cells damaged by DNA polymerase inhibitors or ionizing radiation [2], [21], [45], [46]. We found that yeast Rif1 appears to move with resection forks driven by the Sgs1 helicase [18]; other studies showed that vertebrate Rif1 associates with the Sgs1-homologue, the BLM helicase, at replication forks [47]. Whether Rif1 and Sgs1 are acting in the same pathway is discussed in Text S1, Figure S1.

In conclusion, Rif1 has important and most likely conserved roles, inhibiting the checkpoint-dependent responses to DNA damage (ssDNA accumulation). Consequently, Rif1 permits cells to proliferate with DNA damage, which is a pre-requisite for chromosomal instability. Moreover, Rif1 is the first protein shown to inhibit the recruitment of RPA to ssDNA; through this effect, Rif1 could modulate important RPA-dependent processes, for example DNA replication. Further experiments will be required to understand all the consequences of the Rif1-effect in yeast and mammalian cells.

## Methods

### Yeast strains

All strains are derivates of W303 and are *RAD5^+^*. The *cdc13-1 rif1Δ* and *cdc13-1 rif2Δ* strains were generated by transformation of DLY1230 with PCR products using pFA6a-kanMX6 as a template [48]. *RIF1-MYC* strains were generated in the same way, using pFA6a-13Myc-KanMX6 as a template [48]. The last 1695 nt of the *RIF1* gene were deleted to generate strains with a Rif1 C-terminus deletion (Rif1-C*Δ*). To over-express Rif1, we replaced the 0.5 kb genomic DNA upstream of the ATG of *RIF1* with the *GAL1* promoter, using pFA6a-kanMX6-*PGAL1* and pFA6a-kanMX6-*pGAL1-GFP* as templates [48]. Other strains were obtained by genetic crosses between *cdc13-1 rif1Δ* and the following strains: *HA2-DDC1* (YLL334 [49] and *DDC2-YFP RFA1-CFP* (W3924-11B [50]).

### Cell culture, serial dilution, and cell cycle analysis

The YPD medium (Yeast extract, Peptone, and Dextrose) was supplemented with adenine at 50 mg/l. For experiments testing the maximum permissive temperature, cells grown overnight at 20°C were diluted to about 1.5×10^7^ cells/ml, followed by 5-fold dilution series set up in 96-well plates. Small aliquots were transferred to YPD plates using metal prongs. Plates were incubated for 2.5 days at the indicated temperature. For Rif1 over-expression experiments, cells were grown in YPR medium (YP with 2% raffinose), followed by a 5-fold dilution series and transfer to YPD or to YPG plates (YP with 2% galactose). Cell cycle analysis was performed by fluorescent microscopy, after staining samples with DAPI and sonicating them, to separate individual cells. Following fractions were counted: cells without buds (in the G1 phase), with small buds (in the S-phase), with large anucleated buds (at the G2/M transition) or with nucleated buds (in anaphase/telophase). Wild type cells are equally distributed between these stages of the cell cycle, if growing exponentially.

### Single-stranded DNA (ssDNA) measurements

Single-stranded DNA (ssDNA) measurements at sub-telomeric and single gene loci were performed by QAOS as previously described, except that we used asynchronous populations of cells cultivated at the indicated temperatures [29], [51]. Genomic DNA was extracted, purified and quantified at a centromere-proximal location (*PAC2*). SsDNA was quantified by QAOS within the Y′ sub-telomeric repeats (at about 1 kb from chromosome ends) and at the following single gene loci from the right arm of chromosome V: *YER188W*, *YER186C* and *PAC2*. Taqman primers and probes used for QAOS were previously described in [29], [51]. Single-stranded DNA measurements in the TG-telomeric repeats were performed using the fluorescent in-gel hybridization assay (FIGA) described in [32], with modifications (described next). Phenol-extracted DNA samples were diluted to about 800 ng/µl, digested with Xho1 and hybridized over night with a CA-rich fluorescent 5′ [CY5]CCCACCACACACACCCACACCC probe (Sigma). In the morning, a fraction of the digested and labelled DNA was denatured for 2 min at 100°C in a total volume of 30 µl and then chilled on ice for 1 h. This denaturing time is optimized for DNA up to 8 µg. DNA was exposed to gel electrophoresis to separate telomeric fragments and then scanned using a Typhoon Trio imager (GE Healthcare Fluorescent). Denatured and native DNA samples were scanned simultaneously, to ensure identical parameters/conditions of detection (leading to more realistic results following normalization of native to denatured DNA signals). The intensity of the signal in each sample was quantified on the original image generated by the Typhoon Trio imager, using the ImageJ (NIH) software. The percentage of ssDNA was calculated as the signal given by native telomeres (minus the immediately adjacent background) normalized to the signal given by denatured telomeres (minus the immediately adjacent background) and multiplied by 100. Additionally, DNA was stained with SYBR Safe for informative purposes.

### Protein extraction and western blotting

Protein extracts were prepared by a trichloroacetic acid (TCA) method described in [52]. For western blotting, proteins were separated on SDS-PAGE and transferred to nitrocellulose membranes (GE healthcare). Membranes were blocked in 5% TBST, incubated with antibodies and analyzed by LAS-3000 (Fujifilm). We used the following antibodies: mouse monoclonal anti-Myc (sc-40, Santa Cruz), mouse monoclonal anti-GFP (11814460001, Roche), rat monoclonal anti-HA (11867423001, Roche) and goat polyclonal anti-Rad53 (sc-6749, Santa Cruz), anti-Sgs1 (sc-11993, Santa Cruz). Secondary antibodies included rabbit anti-mouse (ab6728, Abcam), donkey anti-goat (sc-2020, Santa Cruz) and rabbit anti-rat (ab6734, Abcam).

### Chromatin immuno-precipitation (ChIP)

Chromatin immuno-precipitation (ChIP) was carried out by standard methods [53], [54].The association of Rif1-MYC, Ddc1-HA, Ddc2-YFP, Rpa1-CFP and Rif1-HA with chromatin was detected using antibodies (described above) directed against the respective tags; YFP and CFP were detected with anti-GFP antibodies. The association of Rad9 and Rap1 was detected with anti-Rap1 (sc-6663, Santa Cruz) and anti-Rad9 (sc-50442, Santa Cruz) antibodies, tested for their specificity by western blotting. RPA was also detected with specific antibodies PAB13584 (Abnova). Additionally, cells were treated with anti-goat antibodies (sc-2033, Santa Cruz) to assess the background cross-linking. For each time point, the background normalized to the input was subtracted from the immuno-precipitated DNA, also normalized to the input. Input, immuno-precipitated DNA and background were quantified by real-time PCR (StepOne Plus, Applied Biosystems) using genomic DNA standards.

## Supporting Information

Figure S1 (a-b) Rad24 and Sgs1 do not affect the association of Rif1 with DNA damage; (c) Sgs1 and Rif1 affect proliferation of *cdc13-1* cells differently; (d) Rif1 does not affect the amount of checkpoint proteins in cells. (a–b) Association of Rif1 and RPA with *YER188W* in *cdc13-1*, *cdc13-1 rad24Δ* and *cdc13-1 sgs1Δ* strains, after 5 h incubation at 36°C. The reduced Rif1-association in *cdc13-1 rad24Δ* and *cdc13-1 sgs1Δ* versus *cdc13-1* strains in (a) corresponds to reduced RPA-association in (b), therefore it can be explained by reduced DNA damage. (c) Growth of serial dilution of *cdc13-1* cells with or without additional mutations (indicated at the right of each row) at different temperatures, indicated above each plate. (d) Western blot detection of checkpoint proteins (indicated on the right) in *cdc13-1 RIF1+* cells (left half of the picture) and *cdc13-1 rif1Δ* cells (right half). Cells were grown at 21°C (time 0) and then incubated for 160 min at 27°C; samples were collected every 20–40 min.
(EPS)

Text S1 Genetic interactions between *RIF1* and *SGS1* during telomere uncapping.
(DOC)
