# Supplementary material for: A Novel Checkpoint and RPA Inhibitory Pathway Regulated by Rif1
Source: PLoS Genet. 2011 Dec 15;7(12):e1002417. doi: 10.1371/journal.pgen.1002417 (PMC3240596; doi:10.1371/journal.pgen.1002417)
Supplement: Text S1 — Genetic interactions between RIF1 and SGS1 during telomere uncapping. (DOC) [file pgen.1002417.s002.doc]

**Text S1**

A recent study by Xu et al reported that: 1) vertebrate Rif1 works in the same pathway as the helicase BLM (the homologue of yeast Sgs1) to maintain normal DNA replication; 2) yeast Rif1 may work in a different pathway to Sgs1 at stalled replication forks [1]. The reason for this difference between vertebrate and yeast Rif1 was proposed to be the inability of yeast Rif1 to bind DNA [1]. In our study, we showed by chromatin immuno-precipitation that yeast Rif1 associated with several chromosome regions and co-localized with Sgs1 (Figure 1). However, Rif1 did not affect the association of Sgs1 with DNA damage (Figure 3 f-h). Conversely, we asked whether Sgs1 affected the association of Rif1 with DNA damage. To test this hypothesis, we incubated *cdc13-1* cells (+/- an *sgs1∆* mutation or a *rad24∆* mutation) for 5 hours at to 36^o^C, to induce telomere uncapping. Rad24^Rad17^ is a checkpoint loading clamp and DNA processing factor. After 5 h, we tested by chromatin immuno-precipitation whether the association of Rif1 with the *YER188W* locus was affected by an *sgs1∆* or a *rad24∆* mutation. We found that about 30-50% less Rif1 accumulated in the absence of Sgs1 or Rad24 (Figure S1a).

However, less Rif1 could be explained by less DNA damage in the absence of Sgs1/Rad24, since these proteins are involved in generating ssDNA [2, 3]. We tested this hypothesis by detecting the association of RPA with DNA in the same samples we detected Rif1. We found reduced RPA levels in the absence of Sgs1 and Rad24 (Figure S1b), indicating reduced ssDNA levels. Therefore, reduced Rif1 levels in the absence of Sgs1 and Rad24 correlated with reduced DNA damage. We conclude that Sgs1 and Rad24 are not required for the association of Rif1 to DNA damage, similarly to the finding that BLM was not required for the association of vertebrate Rif1 to DNA damage [1].

We also determined whether Rif1 and Sgs1 genetically interact during telomere uncapping, by testing how they affected the temperature-sensitivity of *cdc13-1* cells. We found that unlike a *rif1∆ * mutation, an *sgs1∆* mutation did not increase the temperature-sensitivity of *cdc13-1* cells; instead an *sgs1∆* mutation caused poorer growth (smaller colonies) at all tested temperatures (Figure S1c). Moreover, Rif1 was required for growth of *cdc13-1* cells at 23-25^o^C, irrespective of Sgs1. No additive effects on the proliferation of *cdc13-1* cells could be detected for Rif1 and Sgs1; however, this does not imply that Sgs1 and Rif1 act in the same DNA damage response pathway. Since *cdc13-1 sgs1∆* cells have less damage at chromosome ends (Figure S1b) consistent with [3], the fact they form smaller colonies suggests they accumulate a different type of damage, for example a DNA replication-dependent damage. Therefore, one plausible explanation for a lack of additive effects for Rif1-Sgs1, could be that *sgs1∆* cells have to go through several rounds of DNA replication to accumulate the damage that makes them grow poorly, whereas this accumulation of damage will be prevented if cells arrest in G2/M, due to a lack of Rif1.

Interestingly, *rif1Δ* and *sgs1∆* mutants affected growth of *cdc13-1* cells in a similar way to how their vertebrate homologues affected growth of cells treated with HU in Xu et al [1]. We believe that a lack of additive effects is not sufficient to claim that Rif1 and BLM act in the same pathway, though. As in the case of their yeast homologues, other interpretations could be possible: since Rif1 inactivation has clearly the strongest effect, it may prevent the effects of a BLM inactivation from manifesting when both genes are inactive.

**References:**

1. Xu D, Muniandy P, Leo E, Yin J, Thangavel S et al (2011) Rif1 provides a new DNA-binding interface for the Bloom syndrome complex to maintain normal replication, Embo J. 29: 3140-55.

2. Zubko MK, Guillard S, Lydall D (2004) Exo1 and Rad24 differentially regulate generation of ssDNA at telomeres of Saccharomyces cerevisiae cdc13-1 mutants, Genetics. 168: 103-15.

3. Ngo HP, Lydall D. (2010) Survival and growth of yeast without telomere capping by Cdc13 in the absence of Sgs1, Exo1, and Rad9, PLoS Genet. 6: e1001072.
